# Using Machine Learning to Predict Swine Movements within a Regional Program to Improve Control of Infectious Diseases in the US

**DOI:** 10.3389/fvets.2017.00002

**Published:** 2017-01-19

**Authors:** Pablo Valdes-Donoso, Kimberly VanderWaal, Lovell S. Jarvis, Spencer R. Wayne, Andres M. Perez

**Affiliations:** ^1^Department of Veterinary Population Medicine, College of Veterinary Medicine, University of Minnesota, St. Paul, MN, USA; ^2^Department of Agricultural and Resource Economics, University of California Davis, Davis, CA, USA; ^3^Veterinary Services Pipestone, Pipestone, MN, USA

**Keywords:** swine industry, pig movements, regional control programs, Minnesota, random forest, social network analysis

## Abstract

Between-farm animal movement is one of the most important factors influencing the spread of infectious diseases in food animals, including in the US swine industry. Understanding the structural network of contacts in a food animal industry is prerequisite to planning for efficient production strategies and for effective disease control measures. Unfortunately, data regarding between-farm animal movements in the US are not systematically collected and thus, such information is often unavailable. In this paper, we develop a procedure to replicate the structure of a network, making use of partial data available, and subsequently use the model developed to predict animal movements among sites in 34 Minnesota counties. First, we summarized two networks of swine producing facilities in Minnesota, then we used a machine learning technique referred to as random forest, an ensemble of independent classification trees, to estimate the probability of pig movements between farms and/or markets sites located in two counties in Minnesota. The model was calibrated and tested by comparing predicted data and observed data in those two counties for which data were available. Finally, the model was used to predict animal movements in sites located across 34 Minnesota counties. Variables that were important in predicting pig movements included between-site distance, ownership, and production type of the sending and receiving farms and/or markets. Using a weighted-kernel approach to describe spatial variation in the centrality measures of the predicted network, we showed that the south-central region of the study area exhibited high aggregation of predicted pig movements. Our results show an overlap with the distribution of outbreaks of porcine reproductive and respiratory syndrome, which is believed to be transmitted, at least in part, though animal movements. While the correspondence of movements and disease is not a causal test, it suggests that the predicted network may approximate actual movements. Accordingly, the predictions provided here might help to design and implement control strategies in the region. Additionally, the methodology here may be used to estimate contact networks for other livestock systems when only incomplete information regarding animal movements is available.

## Introduction

Between-farm direct or indirect contact *via* movement of animals or biological materials (e.g., semen), or cross-contamination through inputs such as machinery or human workers, is among the most important factors contributing to disease spread in food animals (1). Farm-to-farm contacts spread diseases that affect the US swine industry, including porcine reproductive and respiratory syndrome (PRRS) and porcine epidemic diarrhea (PED). For both PRRS and PED, animal movements (e.g., gilts, boars, weaned pigs, feeder pigs, and cull animals) represent one of the most important disease transmission routes between farms (2–6).

Understanding the network structure of food animal industries is critical for efficient production and disease control. For example, the sharing of information among agents (e.g., farmers, suppliers, and brokers) within a network may result in an increase of economic efficiency due to the selection of strategies that can decrease production and/or transaction costs (7). Indeed, social network analysis (SNA) is an analytical tool that has been widely used in the field of veterinary medicine to design disease control plans (8). SNA has been used to quantify the nature of connections (referred to as *edges* or *contacts*) among elements (*nodes* or *vertices*) in a population (9). Nodes may be farms or other facilities (e.g., slaughter houses, truck wash disinfection stations, or feed plants) from, to, or through which, animal populations are connected, and contacts among nodes may be categorized as direct or indirect (8, 10). SNA enables researchers to better understand animal movement patterns and, consequently, provide insights on how diseases diffuse in a given industry (11–13). For example, in many livestock industries, a minority of farms typically account for the majority of animal movements (14–16). Identification of those few farms, often referred to as “hotspots” or “super-spreaders” for disease transmission, may help formulating contingency plans to control high impact diseases, as timely intervention to targeted farms may enhance the probability of such plans being successful (13–15, 17). Similarly, efforts to improve animal management and biosecurity in super-spreaders may also contribute to reducing disease risk and prevalence (17, 18).

The US swine industry is characterized by large numbers of documented pig movements within and between states and regions. From 1970 to 2001, the number of pigs moved from one state to another (or from Canada to the US) increased from 30 to 50 million (19). Increases in the number and distance of movements reflect growth in the number of farms specializing in specific phases of the production cycle. Indeed, a growing proportion of feeder and finishing swine farms are located in the Midwest in close proximity to the grain used to feed pigs (20). In contrast, breeding populations tend to be located in areas distant from the major growing pig regions, such as the southeastern US, where grain inputs are not as critical (20–22). Whereas the regional specialization of different industry components has undoubtedly improved efficiency, the necessary movement of animals between the two regions alters the risk of long-distance disease spread (1, 22–24).

Animal movements are only partially regulated in the US, and no source provides complete information on such movements. For example, the United State Department of Agriculture, through the animal disease traceability program, collects information on movements of cattle, bison, equines, sheep and goats, swine, and poultry, only when movements cross state boundaries, except when livestock are moved to slaughter facilities or chicks moved from hatcheries (25). The lack of movement data creates a particular problem for the control of diseases, such as PRRS. In that context, regional control programs (RCPs), voluntarily organized and coordinated by producers, serve as means to share sanitary status information among farmers located in a given area. Sharing information within an RCP in Minnesota (RCP-N212) has been correlated with a decrease in PRRS incidence (26), and thus, one may hypothesize that sharing additional information about pig movements would further improve control program effectiveness. Unfortunately, lack of information about between-farm movements hinders attempts to describe network structure, hence impairing ability to prevent and control disease.

To elucidate the role of network structure in the spread of swine diseases, the relation between PRRS manifestation and animal movements between farms (and other related sites, such as buyer stations or market sites) was assessed in two counties in Minnesota (27). A positive association between positive PRRS status and the number of direct and indirect suppliers (in-reach degree) was observed in one county, but no additional network measures were significantly correlated with positive PRRS status (27). Although that early study provided valuable insights about pig movements between sites and their potential contribution to disease spread, a more complete assessment of the structure of contacts is required to understand disease spread. We use data from Wayne (27) and more recent data collected by the RCP-N212 to build a predictive movement model between sites, which is then used to estimate a complete movement network for the RCP-N212 in Minnesota. The results may be incorporated into a disease-spread model to help explain disease dynamics and support disease prevention and control activities within the RCP-N212.

## Materials and Methods

### Data Sources

We used two complementary sets of data to construct our model. The first dataset, referred to as the network building data set, included information on pig movements related to two counties being used to fit the model, whereas the second dataset included information on sites located within the broader RCP-N212 area, which was used for prediction purposes. The first dataset included information collected in two counties that were geographically located within the boundaries of the second dataset; however, the two datasets were collected separately. The first data set was based on surveys conducted with owners, managers, and veterinarians on farms and at market sites located in Stevens and Rice counties, Minnesota in 2006 (27). Animal movement data included origin and destination of sites in and out of Stevens and Rice, geographic locations, and the production type of sites and owner. Production types included boar stud (BS), farrowing (Fa), nursery (N), finishing (Fi) farms, and market sites (M). This last type encompasses buying stations or slaughter plants. Two networks were described in the building dataset, one for each county, i.e., a Stevens network (SN) and a Rice network (RN). Each network contained data on directional animal movements between any given site located within the county and a number of sites located either inside or outside the county.

The second data set, referred to as RCP-N212, contained information on geographical location, owner, and type of site for premises enrolled in the RCP-N212. This data set contains roughly 38% of total swine premises with 100 or more animals located in Minnesota (28). Data were collected between 2012 and 2015. The RCP-N212 comprised 34 counties in Minnesota, including Stevens and Rice counties (26). The University of Minnesota manages the RCP-N212 data under the terms of an agreement with swine producers that protects the confidentiality of the data.

### Network Description

The structures of SN and RN were described using SNA representing directional flows of animal movements between sites. The site-level connectivity of each network was described using *in-* and *out-degree*, calculated as the number of pig movements received or sent by a specific site to or from other sites. *Betweenness*, defined as the number of directed paths that pass through a given site, when the shortest paths between other pairs of sites are traced (9), was also estimated. Metrics were stratified by site type (e.g., BS, Fa, N, Fi, and M) for SN and RN, and differences in centrality measures between types were analyzed using Kruskal–Wallis tests. To assess the correlation between types of sites in each network, the assortativity coefficient (*r*) for a mixing matrix was used, as defined by elsewhere (29), so that

$$r=\frac{\sum_{i}e_{ij}-\sum_{i}a_{i}b_{i}}{1-\sum_{i}a_{i}b_{i}}$$

where *e_ij_* is the fraction of animal movements in the network that connects sites of type *i* to type *j*, and *a_i_* and *b_i_* are fractions of destination-or-origin, respectively, of a movement that is attached to site type *i*. A value of *r* = 0 indicates no assortative mixing or a random network, while a value of *r* = 1 indicates complete assortativity, i.e., that all movements are between sites of the same production type. Alternatively, if *r* < 0 links are more likely to connect two different types of nodes, which is closer to having a randomly mixed network where links often connect unlike nodes (e.g., different type of sites) (29).

Four metrics at the network level were estimated, namely, (1) *network density*, calculated as the fraction of movements that are present in the network relative to the total number possible; (2) *clustering coefficient*, used as a measure of cohesiveness and defined as the probability that two sites that are linked to a common site are also linked to each other; (3) *diameter*, calculated as the largest distance between two sites in the network, where distance is the shortest path between two sites; and (4) the *mean path length*, calculated as the mean length of the shortest paths connecting two sites (9, 30).

Figures and statistical computations were preformed using R V.3.1.1 (31), including the packages *ggplot2* (32), *maps* (33), *MASS* (34), and *igraph* (35).

### Network Prediction

Due to the inherent attributes of nodes, their dimensional distribution, connection features, etc. predicting networks can be challenging (36, 37). Unsupervised and supervised methods have been used to try to elucidate network structures. Unsupervised approaches seek to assign scores to possible links between nodes mainly based on the neighborhood characteristics of each node and path-distances between nodes. While the first estimates the likelihood of a link between two nodes based on the degree of overlap of their neighbors, the second searches for the shortest path-distance among all possible combinations between nodes (36). For example, the preferential attachment prediction has been used to estimate potential connections of a node given the proportional number of neighbors that it has (38), or the Katz coefficient scores the possible links between two nodes subject to a given length paths (39). While unsupervised methods have been popular in network prediction, they fail to handle network dynamics, the mutual dependence of components, and other features inherent of the network structure (e.g., an unbalanced number of links), thus often leading to unstable performance (37). Among supervised methods, random forest (RF) has shown high levels of classification accuracy compared to others techniques such as bagging (37, 40), so it is the approach used here. Here, information provided by SN and RN was used in a RF model to predict animal movements between sites. After predictions were obtained, parameters were extrapolated to predict movements for the entire number of farms within the RCP-N212.

#### RF Model

Models based on classification trees are built using a single rule or a set of rules for a number of variables that split data to predict possible outcomes. A RF is an ensemble of independent classification trees created from bootstrap samples chosen with replacement from a training data set, in which aggregated estimates from each ensemble generates a final prediction of the probability that a given outcome occurs (40, 41), e.g., a link between two sites. The samples that are not selected as bootstrap samples are called “out-of-bag” (OOB) samples and are used to estimate the error rate. The OOB error rate is reduced by ranking predictors and subsequently removing those considered less important. Calculating the difference in accuracy between models in which predictors are present or removed is used to assess predictor importance. Differences are normalized across all trees generated and then ranked based on accuracy of prediction (40, 41).

Using all sites from SN and RN, we created a new dataset (referred to as RF-data) that contained all possible origin-destination pairs of sites within each network. Per each possible pair of sites, we assigned a dichotomous outcome (yes, no) variable (also referred to as a class variable) indicating whether or not the animal movement has occurred between that pair. We used the geographical location of each site to estimate the pairwise Euclidean distance (kilometers) between farms, and generated a dichotomous variable (yes, no) indicating whether or not each pair had a common owner. Additionally, we generated 25 dummy variables, each denoting a possible pair of site types (e.g., Fa–Fi, Fi–M, BS–Fa, etc.), being 1 if the pair site type combination was true and 0 otherwise.

The effectiveness of model prediction is determined using a portion of the data that has not been used to build and tune the model (40). Thus, we split the RF-data randomly, using 75% of observations to build and tune the model (referred to as the training dataset), and the remaining 25% to test or validate our model (referred to as the testing dataset or validation set). In other words, we used the training dataset to create (i.e., train and tune) the RF model and then used the testing dataset to qualify its performance through a confusion matrix: a two by two table displaying the number of observed and predicted movements reported from the model (Table 1). While there is no widely accepted rule-of-thumb for splitting the data, it is preferred to use a larger amount of information for the training set in order to reduce the variance of the parameter estimates (40). Also, we insured that the training dataset contained the same proportion of class variables (yes and no) as the original RF-data by using a data partition function executed by the *caret* package (42) in R (31).

**Table 1:** **Confusion matrix for the class variable (i.e., animal movements = yes or no)**.

Predicted	Observed		Total
	Yes	No	
Yes	a	b	a + b
No	c	d	c + d
Total	a + c	b + d	*N*

*Cells indicate the number of true positives (a), false positives (b), true negatives (d), and false negatives (c)*.

On the other hand, because we anticipated that RF-data would be unbalanced (i.e., only a small fraction of observations were class variable “yes”), a *post hoc* down-sampling approach was implemented to balance the data, i.e., we used a sample that has roughly the same proportion of each outcome class. The down-sampling technique is an efficient way to improve predictions, particularly when using bootstrap samples, given that no information is lost during the process (40, 43). We used a wrapper provided by the *train* function in the *caret* package (42) in R (31) to improve model consistency and to determine the desired standard resampling and performance testing (40, 44). We ran and tuned the RF model using 1,500 trees for each training dataset (unbalanced and balanced), and we implemented 10-fold cross-validations to estimate and rank the most important predictors.

Subsequently, we compared performance comparing predictive (or expected) versus observed movements for both, unbalanced and balanced testing datasets by using their confusion matrixes. As result, we compared the accuracy, Kappa statistic, specificity, sensitivity, and the area under the receiver-operating characteristic (ROC) curve. The ROC curve is a graphical method to test predictive performance by contrasting true positive and negative values. The accuracy rate $\left(\text{AR}=\frac{a+d}{N}\right)$ was used to measure the agreement between the predicted and the observed classes, although AR does not provide any information on the type of error the model is producing. The Kappa statistic (κ) was used to quantify the relation between observed $\left(O=\frac{a+d}{N}\right)$ and expected accuracy $\left(E=\frac{\left(\left(d+b\right)*\left(d+c\right)\right)+\left(\left(c+a\right)*\left(b+a\right)\right)}{N^{2}}\right)$, so that $\kappa =\frac{O-E}{1-E}$ serves as a proxy for model performance (40). Sensitivity $\left(\text{Se}=\frac{a}{a+c}\right)$ and specificity $\left(\text{Sp}=\frac{d}{b+d}\right)$ were used to measure the capability of the model to predict true movements (i.e., “yes”) and non-movements (i.e., “no”), respectively, whereas the area under the ROC (AUC) was used to assess the trade-off between increasing sensitivity and decreasing specificity or vice versa. With RF, the final prediction as to whether movement occurs between a pair of sites (i.e., class variable = yes) is based on a given probability threshold (i.e., 0.5). We tested varying threshold probabilities (i.e., ≥0.5) to maximize the κ value.

Our RF model utilized a data set of 14,307 observations (75% of all observations) and 28 variables, thus complexity of the algorithm is given by *O*(*v* × *n*log(*n*)), where *v* is the number of variables and *n* is the number of observations. This analysis took around 45 min to complete on a standard MacBook Pro^®^, though other packages such as ranger and random jungle may achieve faster performances for larger data sets and down-sampling can further optimize run times (45).

Finally, using the observed and predicted animal movements, we conducted an SNA to contrast centrality measurements between the observed and predicted network using the non-parametric Kruskal–Wallis test.

#### Prediction of the RCP-N212 Network

We used our final model to predict animal movements among sites in the RCP-N212. Using data from RCP-N212, we generated all possible pair combinations among sites located within that RCP area. Similar to the analyses performed with the RF-data, we estimated Euclidean distances (kilometers) between each pair of sites and generated a dichotomous (yes, no) variable for ownership and 25 dummy variables for possible combinations of types of sites. Acknowledging that movements of animals must also occur to and from sites located out of the RCP-N212 and to avoid overestimations in the number of movements occurring within sites in the RCP, we restricted the number of predicted movements among sites in the RCP-N212 using the maximum values of *in-* and *out-degree* per each type of site observed in SN and RN.

We summarized the distributions of centrality measures of the predicted network for the entire RCP-N212 and for each of its 34 counties. We used the same metrics as described in Section “Network Description” at site and network levels. We performed a spatial analysis of centrality measures using a 2D-kernel density estimation. This allowed us to evaluate the intensity of pig movements (e.g., to, through, and from other sites) in a given unit of space by approximating its probability density function (34, 46, 47).

## Results

### Network Description

**Figure 1:**
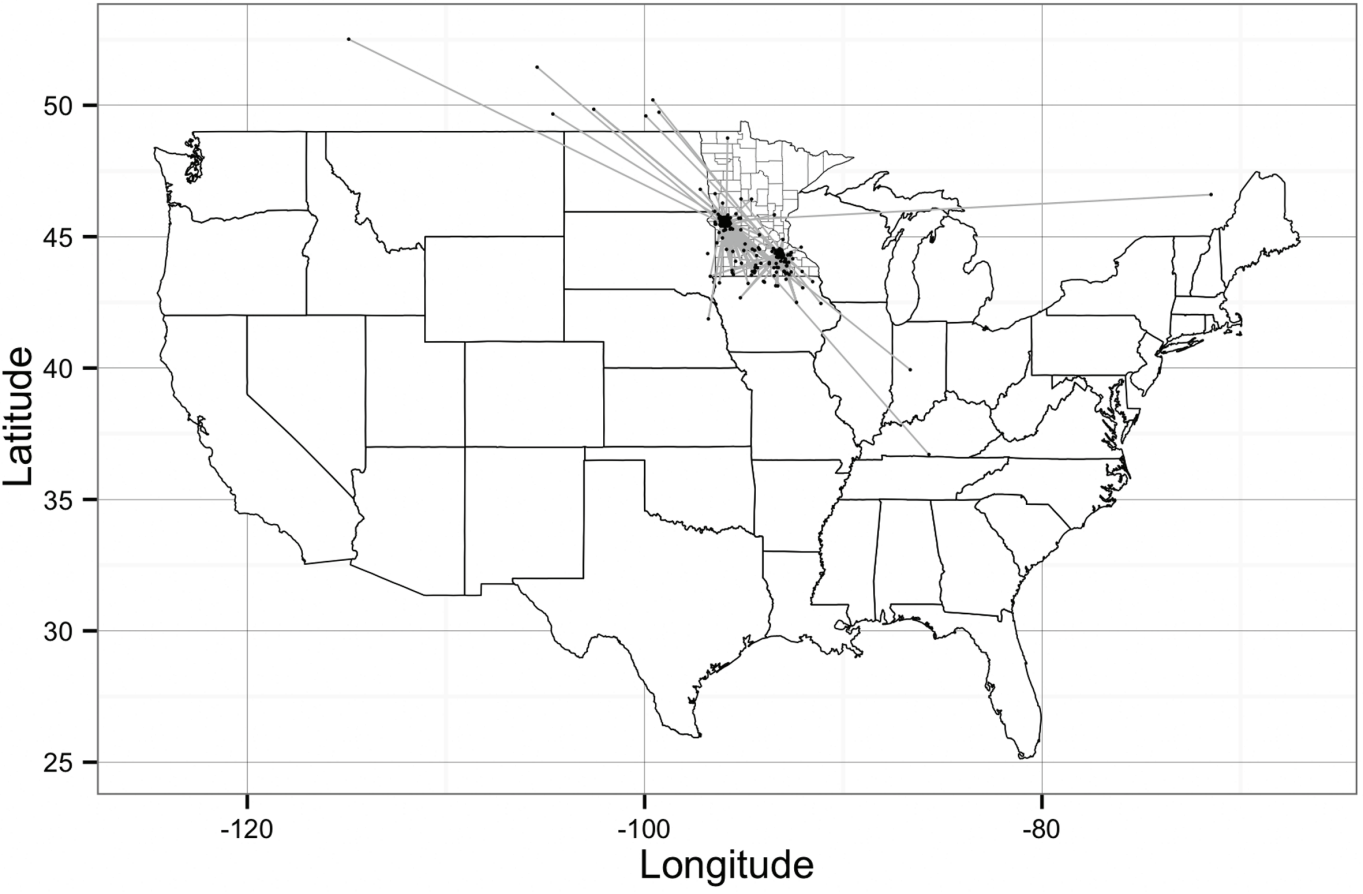
**Geographical representation of the Stevens network and Rice network of animal movements between swine farms and/or market sites**. Dots represent geographical location of sites and straight gray lines represent animal movements. [Source: Wayne (27)].

The network building data set included 237 sites (220 farms and 17 market sites), of which 33 and 19% were located within Stevens County and Rice County, respectively. The remaining 48% of sites were not located within those counties, but involved animal movements to or from Stevens and Rice (Table 2), some covering long distances (Figure 1). We identified 474 animal movements (286 movements in SN and 215 movements in RN), some of which connected the two networks (Table 2). Only 14% of all site types located in Stevens or Rice had movements with sites located in the other county, and all these were movements from finishing farms to market sites.

**Table 2:** **Number of sites by production type and network**.

Network	BS		Fa		N		Fi		M		Total	
Rice network	0	(0)	21	(8)	18	(7)	52	(28)	6	(1)	97	(44)
Stevens network	3	(1)	43	(20)	12	(7)	43	(23)	6	(1)	107	(52)
Both	0	(0)	0	(0)	0	(0)	28	(27)	5	(2)	33	(29)
Total	3	(1)	64	(28)	30	(14)	123	(78)	17	(4)	237	(125)

*Values in parentheses indicate number of sites inside the noted county*.

*BS, boar stud; Fa, farrowing; N, nursery; Fi, finishing; M, market sites*.

**Figure 2:**
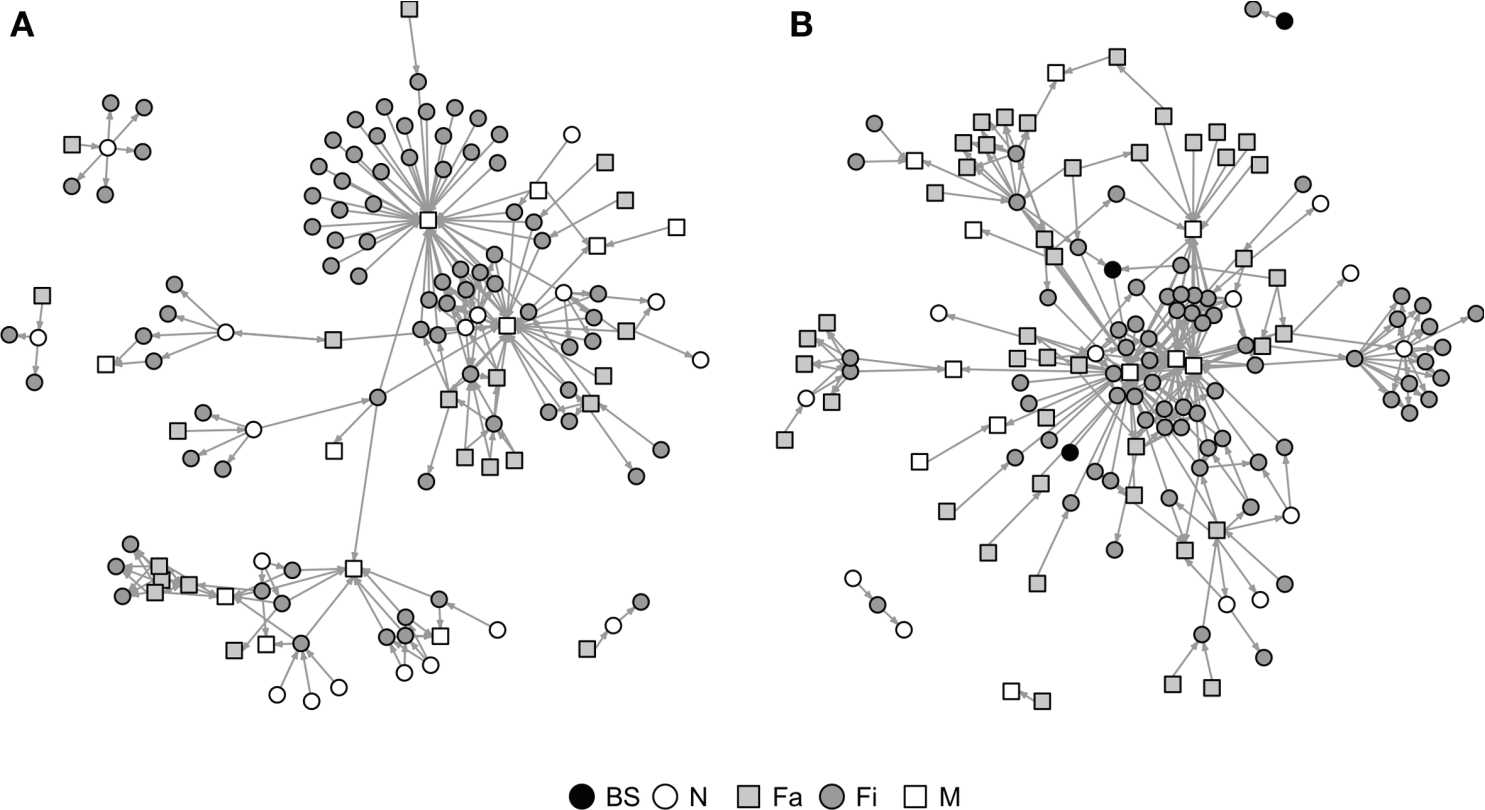
**Graphical representation of Rice (A) and Stevens networks (B)**. Circles and squares represent either swine farms (BS, boar stud; Fa, farrowing; N, nursery; and Fi, finishing) or market sites (M).

Graphical representations of the networks indicate a confluence of paths toward finishing farms and then to market sites (Figure 2). Thus, markets and finishing farms served as hubs in the network. As expected, the most likely movements occurred between sites of different types (*r* = −0.13 and *r* = −0.16 for SN and RN, respectively) that followed downstream flows, i.e., a vertical structure (Table 3). For example, movements from farrowing (Fa) or nursery (N) farms to finishing farms (Fi) were more frequent compared to other possible types of destinations, i.e., market sites (M), boar studs (BS), farrowing (Fa), or nursery (N) farms (Table 3). Markets were the most likely destinations for finishing farms (*e*_FiM_ = 0.40 and *e*_FiM_ = 0.41 for SN and RN, respectively), although finishers also sent pigs into upstream destinations, including nurseries and farrowing farms (e.g., N, Fa, etc.), probably to provide replacement animals (Table 3). The most likely destination for farrowing farms was finishers, followed by nurseries, consistent with the industry trend to eliminate nurseries as midpoint stations (20) (Figure 2; Table 3).

**Table 3:** **Mixing matrix *e_ij_* for type of farm in Rice network (RN) and Stevens network (SN)**.

Network		SN						RN					
Destination		BS	Fa	Fi	M	N	*a_i_*	BS	Fa	Fi	M	N	*a_i_*
Origin	BS	0.00	0.00	0.00	0.01	0.00	0.01	–	–	–	–	–	–
	Fa	0.00	0.03	0.09	0.10	0.04	0.26	–	0.06	0.12	0.05	0.05	0.27
	Fi	0.01	0.10	0.07	0.40	0.00	0.58	–	0.03	0.00	0.41	0.00	0.45
	M	0.00	0.00	0.00	0.02	0.00	0.02	–	0.00	0.00	0.02	0.00	0.02
	N	0.00	0.00	0.13	0.00	0.00	0.13	–	0.00	0.24	0.01	0.00	0.26
*b_i_*		0.01	0.14	0.29	0.53	0.04	1.00	–	–	0.36	0.50	0.05	1.00

*BS, boar stud; Fa, farrowing; N, nursery; Fi, finishing; M, market sites*.

*r_SN_ = −0.13 and r_RN_ = −0.16*.

**Figure 3:**
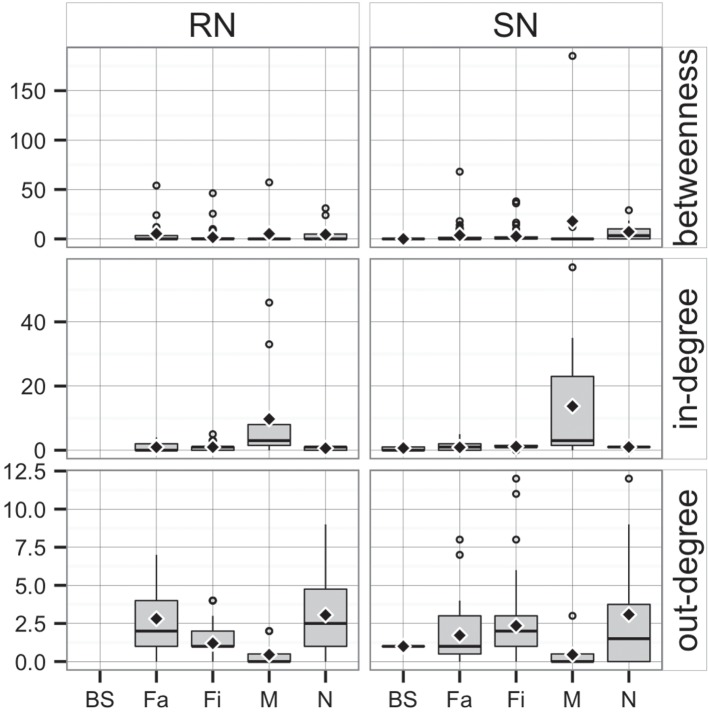
**Boxplot of betweenness, in- and out-degree in Rice network and Stevens network by production type (BS, boar stud; Fa, farrowing; N, nursery; Fi, finishing; M, market sites)**. Boxes indicate the first and third percentile; middle bars represent the median and diamonds represent the mean.

Whereas *in-degree* and *betweenness* were slightly higher in SN than RN (*P* = 0.05 and *P* = 0.04, respectively), there was no statistical difference between the two networks in out-degree (*P* = 0.31). In contrast, *in-degree* varied across production types for both networks (*P* < 0.01 for both), with markets having a higher *in-degree* (mean = 11.7, SD = 16.8, min = 0 and max = 57) (Figure 3). Nurseries exhibited significantly higher out-degree than other production types (*P* = 0.01 for SN and *P* < 0.01 for RN), each shipping animals to three different sites on average, with a maximum of 12. *Betweenness* did not significantly differ across production types within RN (*P* = 0.17) but was statistically different among different types of sites in SN (*P* = 0.02) (Table 4).

**Table 4:** **Summary of centrality measures at site-level using both Stevens network and Rice network**.

Centrality measure	BS	Fa	Fi	M	N
Betweenness	0.00	4.20	2.07	11.56	5.61
	(0)	(1.43)	(0.51)	(8.67)	(1.66)
	0^a^	68^a^	46.21^a^	185.14^a^	31^a^
In-degree	0.67	0.92	1.05	11.73	0.77
	(0.67)	(0.14)	(0.07)	(3.59)	(0.1)
	2^a^	5^a^	5^a^	57^a^	2^a^
Out-degree	1.00	2.08	1.74	0.46	3.07
	(0)	(0.26)	(0.15)	(0.18)	(0.62)
	1^a^	8^a^	12^a^	3^a^	12^a^

Values denote means, SEs in parenthesis, and maximums with superscript “a.”

*BS, boar stud; Fa, farrowing; N, nursery; Fi, finishing; M, market sites*.

Both networks exhibited similar densities, 0.014 for SN and 0.013 for RN. However, RN was relatively more cohesive than SN, as shown by a higher clustering coefficient, revealing a 0.007 and a 0.056 probability, respectively, that two sites moving animals to a common site were also connected to each other. As a result, RN also had a smaller diameter (4) than SN (5), though mean path lengths were relatively similar (1.82 for RN and 1.85 for SN). In turn, the distances between sites varied considerably, from less than 1 to more than 1,000 km. The overall mean distance between sites was 111 km, with nurseries and farrowing farms receiving animals from longer distances and boar studs shipping animals to sites located more than 500 km away (Table 5).

**Table 5:** **Summary of distances (km) between origin and destination by type of site**.

Type destination		BS	Fa	Fi	M	N	Mean
Type origin	BS			1,894.81	20.36		645.18
				(–)	(13.68)		
				1,894.81^a^	34.04^a^		
	Fa		113.71	122.50	32.95	170.83	102.98
			(46.72)	(38.56)	(7.78)	(61.61)	
			700.64^a^	1,582.98^a^	214.84^a^	1,066.67^a^	
	Fi	9.94	181.90	98.16	122.97	21.51	128.49
		7.65	(43.15)	(22.28)	(8.75)	(–)	
		17.58^a^	1285.83^a^	271.21^a^	354.65^a^	21.51^a^	
	M				267.49		267.49
					(49.57)		
					432.79^a^		
	N		126.73	48.98	42.12	22.08	50.21
			(101.77)	(7.73)	(30.68)	(–)	
			228.50^a^	270.83^a^	72.81^a^	22.08^a^	
Mean		9.94	155.76	90.30	109.95	158.91	111.14

Values denote means, SEs in parenthesis, and maximums with superscript “a.”

*SEs could not be calculated in all cases due to small sample size*.

*BS, boar stud; Fa, farrowing; N, nursery; Fi, finishing; M, market sites*.

### Network Prediction

#### Random Forest

There were 19,075 possible pairs for RN and SN. Among them, a minority (2.6%) corresponded to true movements (i.e., class = yes). RF models for both balanced (similar proportion of class variable “yes” and “no”) and unbalanced datasets used 1,500 trees, and the optimal number of predictors (*m*_try_) estimated was 27 and 20, respectively. We observed a higher κ for the unbalanced dataset, indicating a higher accuracy (Table 6). However, use of the unbalanced datasets resulted in predictions that were strongly biased toward the majority class, with the class variable “no” accounting for 97.4% of total pairs.

**Table 6:** **Results of the random forest (RF) analyses for balanced and unbalanced datasets**.

Model	Accuracy	Kappa	Sensitivity	Specificity	Area under receiver-operating characteristic
Balanced RF model	0.88	0.23	0.808	0.883	0.93
Unbalanced RF	0.98	0.34	0.232	0.997	0.85

**Figure 4:**
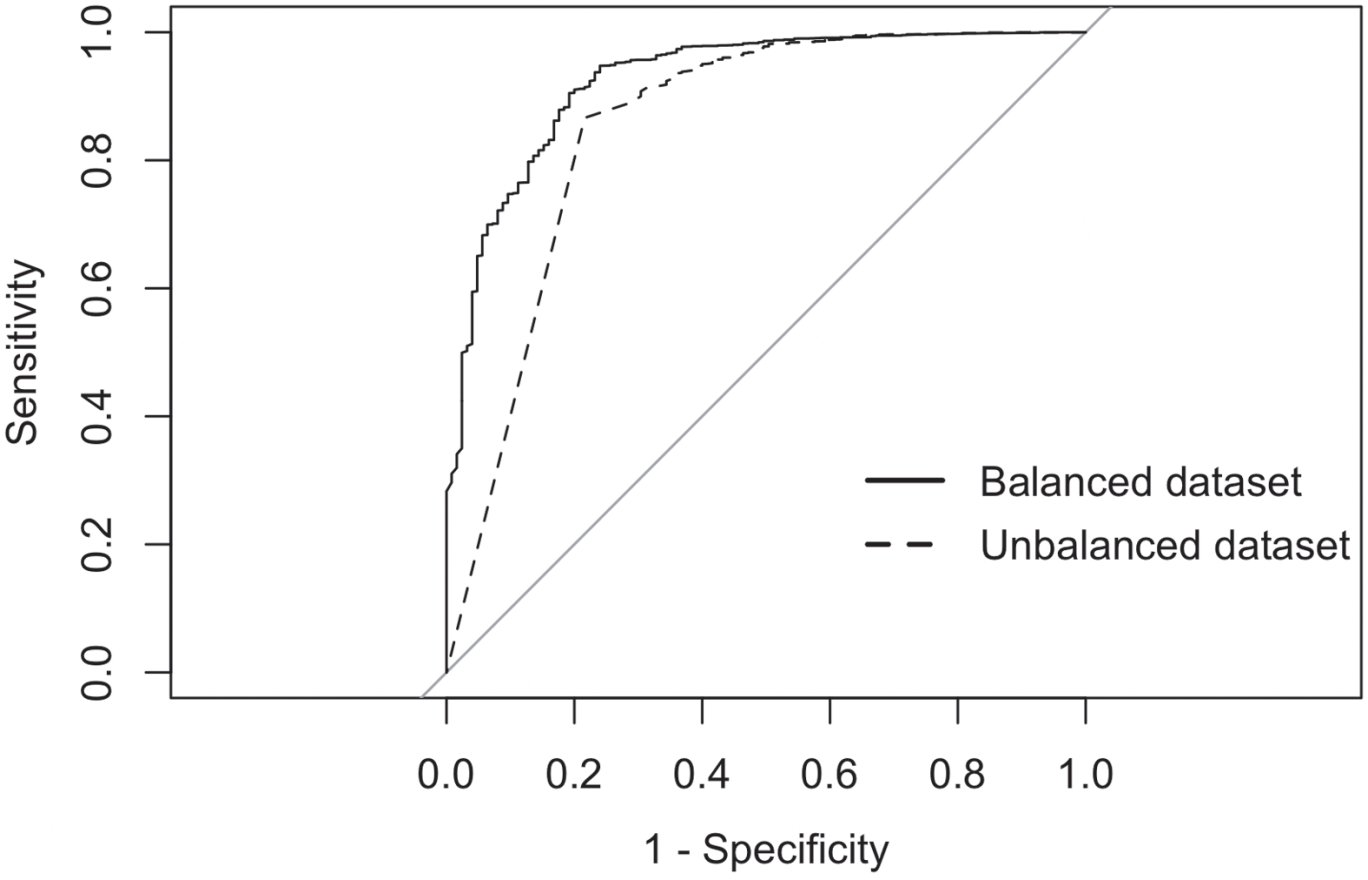
**Receiver-operating characteristic curves for the RF analyses using balanced and imbalanced datasets**.

**Figure 5:**
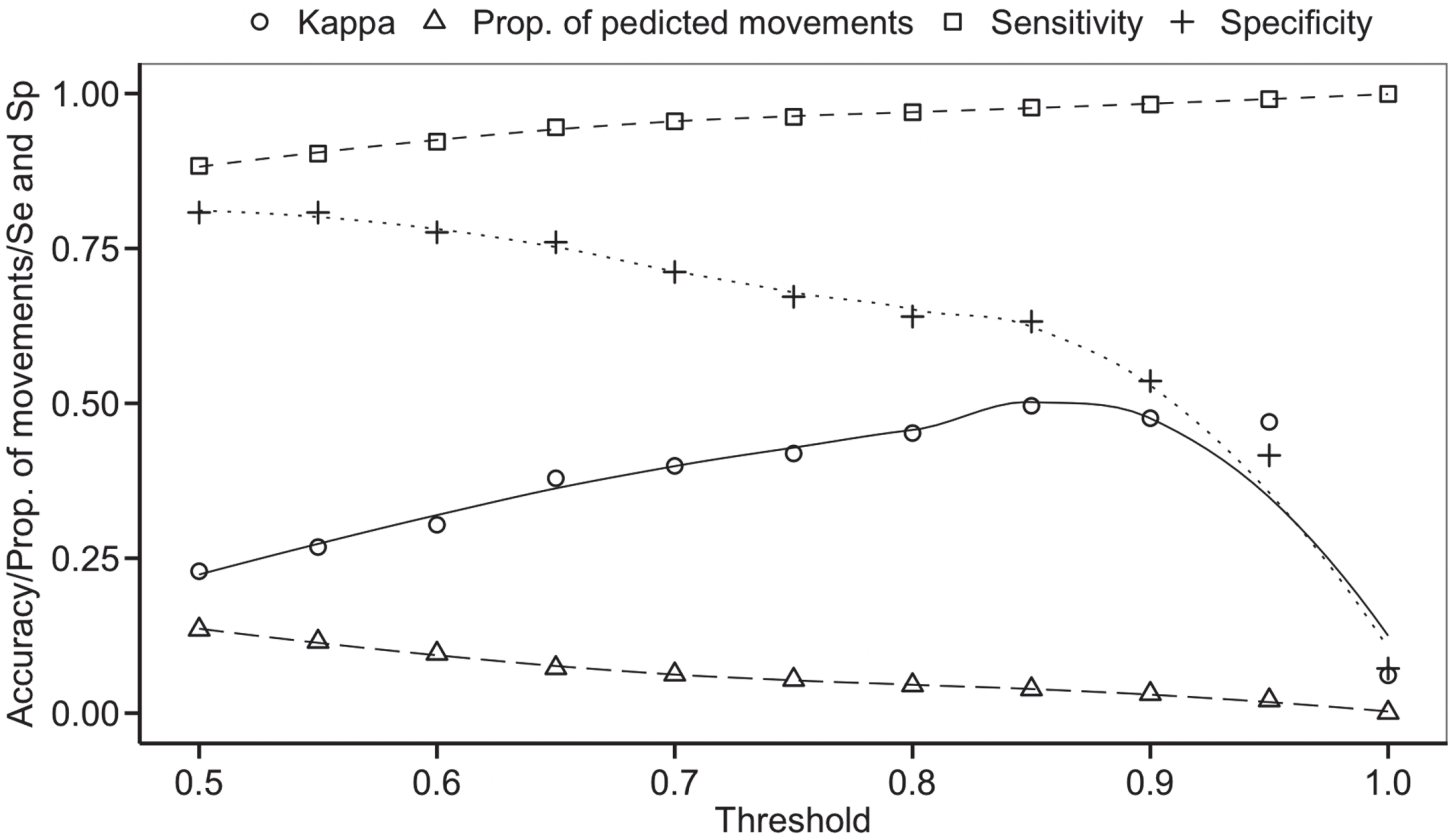
**Kappa statistic accompanying the threshold probability for the proportion of predicting movements out of the total pairs using balanced data**. Sensitivity (Se) and specificity (Sp) are also reported through different thresholds.

**Figure 6:**
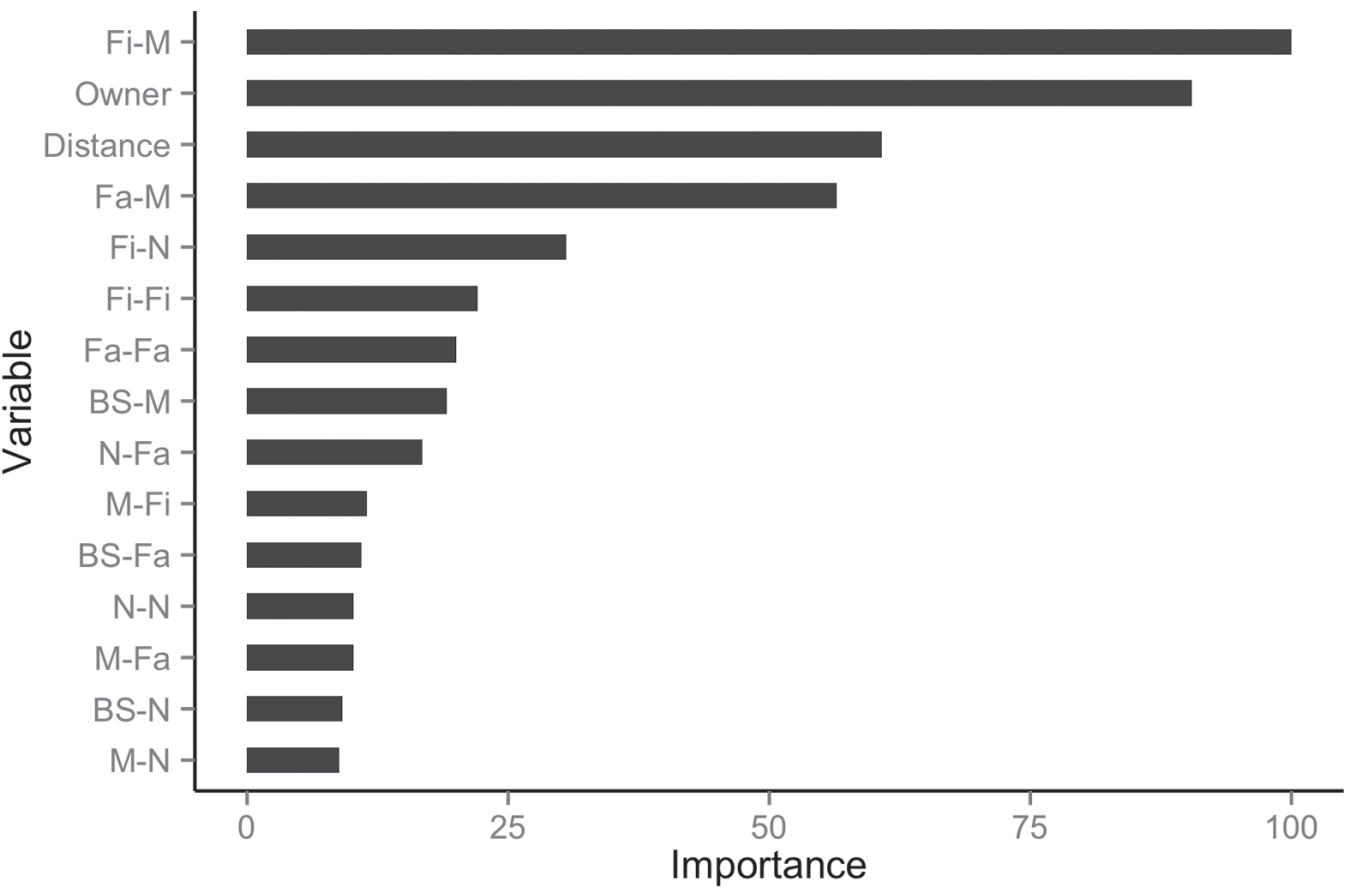
**Rank of the 15 most important variables for network prediction using the balanced dataset**.

The balanced dataset optimized sensitivity with a low penalty to specificity. Moreover, inspection of the AUC indicated that false positives and negatives were minimized with the balanced dataset (Figure 4). However, the 0.5 default probability threshold used by the RF to predict animal movement between a pair of sites (i.e., class variable = yes) resulted in low agreement (i.e., when κ < 0.3) between observed versus predicted movements (40) (Table 6). Increasing the threshold from 0.5 to 0.85 resulted in an increase in agreement (κ = 0.5, Figure 5) between observed and predicted movements. The most important variables predicting movements were farm type (downstream combinations from finishers and farrowing farms to market sites), sharing the same owner, and distance (Figure 6).

**Figure 7:**
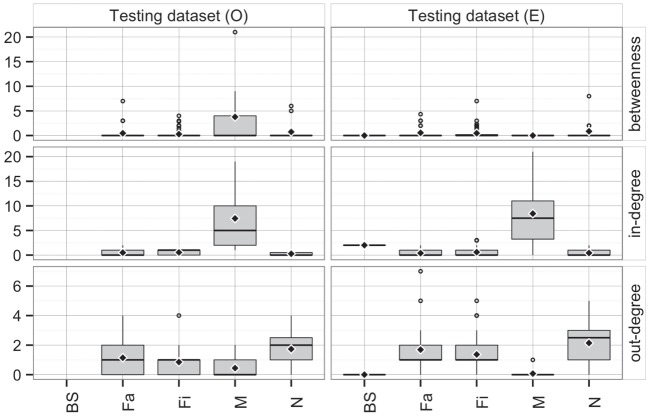
**Boxplot of betweenness, in- and out-degrees in the testing dataset for observed (*O*) and predicted (*E*) movements by type of site**. Boxes indicate the first and third percentile; middle bar represents the median and diamonds represent the mean values.

Comparing the observed (*O*) and predicted (or expected, *E*) networks based on observed and predicted animal movements from use of the *testing dataset*, model predictions provided a reasonable approximation of real movements (Figure 7). Overall, there were no statistical differences between both networks in *betweenness* (*P* = 0.38) and *in-degree* (*P* = 0.97), whereas values for the *out-degree* were significantly different (*P* = 0.02). For the latter, we predicted that, on average, a site would deliver animals to 1.4 sites (SD = 1.3), compared to 1 site (SD = 1.0) observed in the real network.

Furthermore, the patterns of connectivity across farm types were qualitatively similar (Figure 7). Whereas comparisons across production types within observed and predicted networks did not show significant differences in *betweenness* (*P* = 0.32 and *P* = 0.35, respectively), *in-* and *out-degree* were statistically different across production types in both observed (*P* < 0.01 for both) and predicted networks (*P* < 0.01, and *P* = 0.01, respectively). For example, market sites in the observed data received animals from 7.4 different sites, whereas the model predicted receptions from 8.4 different sites. Similarly, whereas the model predicted that a nursery would ship animals into 2.1 farms, observed values indicated 1.7 different farms (Figure 7). On the other hand, there were no significant differences when comparing the observed to predicted centrality metrics by production type (*P* > 0.05) for all, except *betweenness* of market (*P* = 0.02) and *out-degree* of finishing farms (*P* = 0.002).

**Figure 8:**
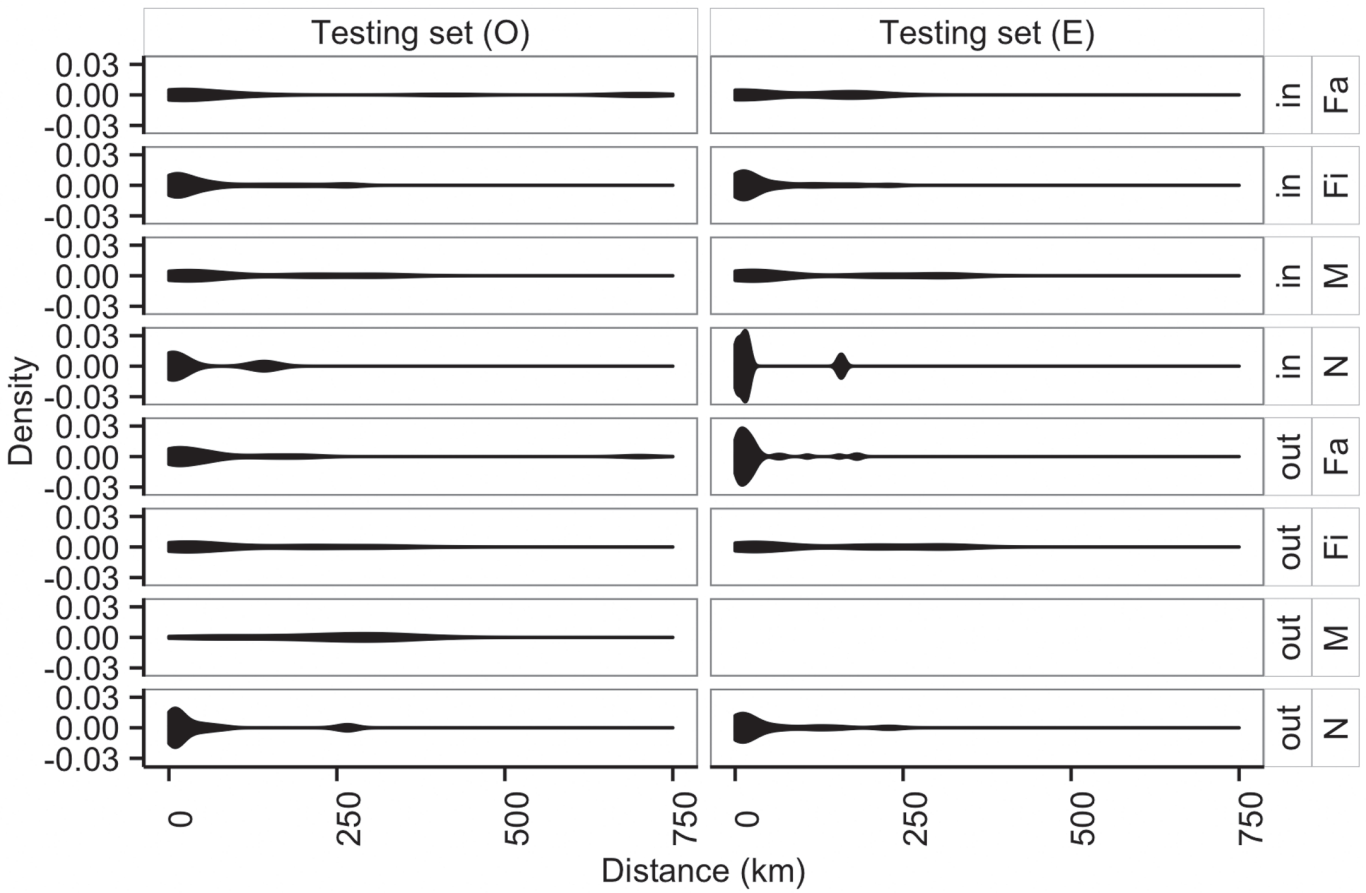
**Distribution of distances (km) of shipments (out) and entries (in) for observed (*O*) and predicted (*E*) movements by type of location**.

Additionally, average distances between observed and predicted movements did not significantly vary across types of sites (N, Fa, Fi, and M with *P*-values of 0.70, 0.05, 0.29, 0.94, respectively) (Figure 8). Among farms, we noticed that finishing farms shipped animals the longest distances (observed and predicted averages 128.4 and 130.7 km, respectively), whereas markets on average received animals from 107.3 km away versus a prediction of 115.7 km.

#### Prediction of the RCP-N212 Network

The RC-N212 dataset contained 830 sites, 65.1% of which specialized in the last stage of production (e.g., growing, finisher, wean-to-finish), and 32% characterized as farrowing farms or nurseries. Only 1.2% of total sites recorded in RCP-N212 were market sites, which were located in only 4 out of the 34 counties in which RCP-N212 sites were located. We generated 688,070 possible origin-destination pairs, and our model predicted that 0.9% of those pairs were likely to move animals between them, using a probability threshold >0.85. However, if the number of likely links for a given farm exceeded the maximum observed *in-* or *out-degree* for its production type (Table 4), the number of contacts was restricted to the maximum degree by randomly selecting from the highly probable links. This process resulted in a network where 0.4% of the total pairs were likely to move pigs between them.

**Figure 9:**
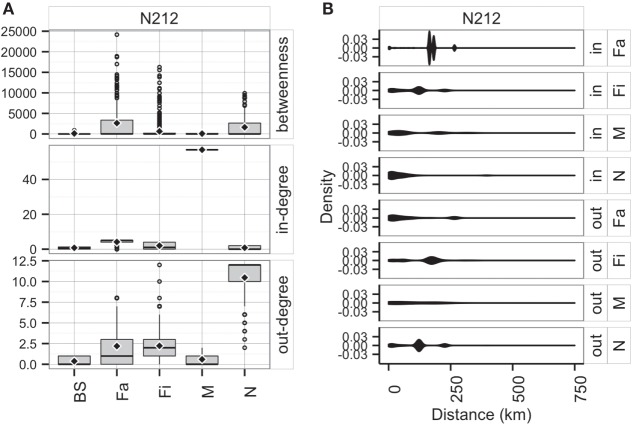
**Boxplot of betweenness, in- and out-degrees distributions (A), and distance (km) distribution of shipments (out) and entries (in) (B) for the predicted network in RCP-N212**.

Unsurprisingly, market sites reached the maximum allowable *in-degree*, receiving pigs from 57 sites, whereas farrowing farms, nurseries, and finishers were expected to receive animals (perhaps replacements), on average, from 4, 1, and 2 sites, respectively. On the other hand, the model predicted that nurseries and farrowing farms would ship pigs (i.e., *out-degree*) to 12 and 10 farms, respectively (Figure 9A). *Betweenness* was highest in farrowing and nursery farms, followed by finishing farms (Figure 9A).

The model using RCP-N212 data predicted animal movement distances that were slightly different from those predicted using the testing dataset presented in the previous section. Finishing farms were expected to ship (i.e., *out-degree*) animals through, on average, 141.4 km (SD = 75.5 km), whereas nurseries, on average, 113.6 km away (SD = 67.7 km) to sites within the RCP-N212. In turn, farrowing farms and market sites were expected to receive animals from longer distances (mean = 168.4 km, SD = 51.0 km, and mean = 104.3 km, SD = 98.5 km, respectively) (Figure 9). The density of the predicted network in RCP-N212 was 0.004, with a clustering coefficient of 2.8%, and a mean path length of 7.26.

**Figure 10:**
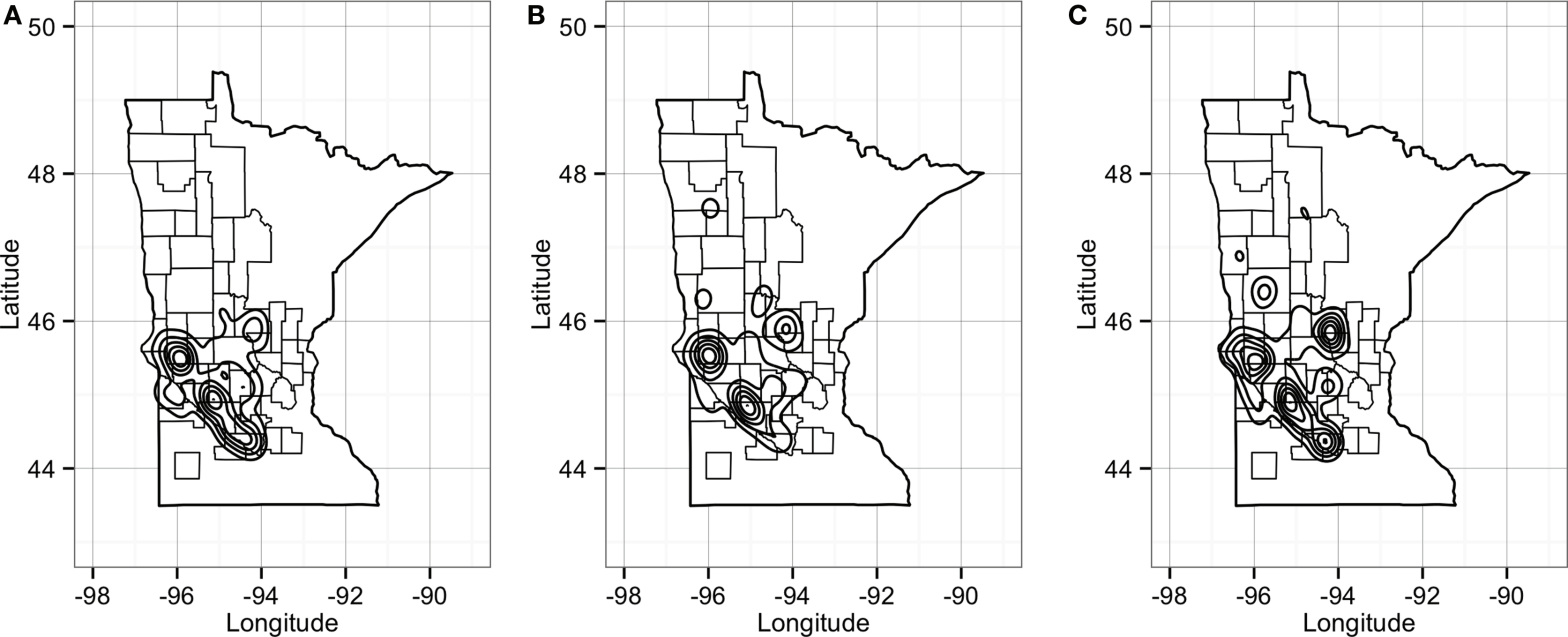
**Plot of geographical density for expected out-degree (A), in-degree (B), and betweenness (C) of sites in the RCP-N212**. Counties delineated compose the area of the RCP-N212.

Predicted pig movements in the RCP-N212 covered large spatial areas, and only 14% were within the same county. In general, most predicted pig movements passed through several counties, with a maximum of 11 counties. Finally, the predicted network for the RCP-N212 suggested a major aggregation of movements to and from sites located in areas toward the southern part of the regional program (Figure 10).

## Discussion

The aim of this research was to predict animal movements among sites located within a given RCP. Unfortunately, data of movement networks are often incomplete or unavailable for food animal industries characterized by a large number of animal movements between sites, such as the US swine industry (19, 25). Therefore, we employed machine-learning techniques to illustrate how models may be fitted by using a subset of the data to increase their completeness and accuracy. Specifically, using information available in only two counties, we studied the likelihood of possible movements among sites in a larger-scale swine disease RCP in Minnesota, referred to as RCP-N212. In general, networks predicted by the RF model were consistent with the observed data used for model training and testing in terms of both spatial and production-type connectivity patterns.

The SN and RN networks exhibited relatively similar centrality patterns and a marked flow of animal movements from upstream to downstream sites in the production chain. However, the network structure of both counties also indicated that finishing (and even market) sites might provide animal replacement (e.g., gilts and boars) to upstream sites. Because outbreaks of diseases, such as PRRS, are also common in downstream sites (26), movements from those sites to upstream sites could perpetuate disease in those areas. Indeed, previous research has shown that despite an overall decrease in the occurrence of PRRS, spatial and temporal aggregations of that disease allowed for continued hotspots throughout the period of study (26). These interactions merit further analysis to explain swine disease dynamics, especially for industry-persistent diseases such as PRRS (2, 6, 48).

While model results suggest that ownership and distance are strongly related to the probability of pig movement between sites, the production type of the origin and destination sites also influenced the probability of pig movements from one location to another. Moreover, if we consider that different types of farms might share transportation services, whereby farms may ship or receive different type of animals (e.g., feeder pigs and finishing pigs), such mixing might facilitate spread disease *via* contaminated vehicles (4, 49). Thus, we suggest that a complete evaluation of disease risks associated with transportation of pigs between facilities should take into account factors such as the commercial relationship between sites, including contractual agreements in the US swine industry (50), and site production type (6).

As mentioned previously, we used information available from two small, county-based networks to estimate parameters for predictions of animal movements that closely fit observed movements as judged by standard statistical tests. We were able to validate our predictions within SN and RN. The parameters generated by our model were used to predict animal movements between sites over a larger area, i.e., RCP-N212. This is essentially an out-of-sample prediction. As data on actual animal movements were not available for RCP-N212, we cannot directly test the accuracy of our predictions for the larger network. The results for larger network appear reasonable in that they are consistent with the topology of the SN and RN networks, and these results may be useful in helping understand actual (but unobservable) animal movements in Minnesota. We believe that such out-of-sample prediction is warranted for the scale of RCP-N212, given that farms within this program are similar to the farms in SN and RN in terms of geography, demography, and management. However, predictions that would rely on more extensive extrapolation (such as at the scale of multiple states) would not be appropriate given the scope of our sampling. In addition, the value of being able to assemble a full network may not be to target individual farms, but rather to capture possible regional patterns in connectivity.

Based on comparisons between observed, county-level data and regional-level model predictions, such as the distribution of movement distances and general attributes of the network, we believe that our predicted full network for RCP-N212 has structural features that are similar to the partial data used to estimate the probability of movement between farms. Thus, our findings appear reasonable and provide insight to better understand animal movement patterns within the RCP-N212. This, in turn, may help farmers design private strategies for sanitary management, as well as aid policy-makers in structure-based decisions. However, given inherent limitations to predictive modeling, we acknowledge that our results may provide only general insights about movement patterns, thus additional work must be done before strong conclusions can be made regarding the utility of the predictions achieved in this way.

The RCP-N212 covers 34 out of 87 counties in Minnesota, accounting for 38% of the total swine facilities with 100 or more heads in the state. Because farm distribution is heterogeneous within Minnesota, with a greater number of farms toward the south (28), it is reasonable to infer that the distribution and type of sites across the RCP-N212 should influence our network predictions. Furthermore, given that 65.1% of the sites are dedicated to the last stage of production, we acknowledge that a fraction of sites within the RCP-N212 must trade animals with sites located in neighboring states, such as Iowa or Illinois, or even more distant states such as North Carolina, where a high number of sow farms are located (20, 51, 52). This could have led to an overestimation of movements between some sites in our model, especially for those underrepresented in the RCP. To tackle this issue, we applied two constraints to our model: (1) we restricted prediction of movements by increasing the probability threshold to 85% when assigning a possible link between two sites and (2) we constrained the maximum number of links per site using maximum values of *in-* and *out-degree*. As a result our movement predictions within the RCP-N212 were conservative, showing a lower density in the expected network (0.4%) than in the two observed networks (1.4% and 1.3% for SN and RN, respectively), although some metrics might have been overestimated, such as *out-degree* for nurseries. This is because there are very few nursery farms in this region, and many of the finishing farms are actually sourcing for pigs from other states outside of RCP-N212. However, our algorithm restricted their choice of nurseries to those within RCP-N212, perhaps leading to a false inflation of their *out-degree*. Future work should expand to larger geographic regions that encompass all stages of production, capturing movements between states. We anticipate that we could improve model predictions by obtaining information regarding contract relationships and animal movements among Minnesota sites and suppliers located outside RCP-N212.

In the predicted RCP-N212 network, we found that sites with higher *in-* and *out-degrees* overlap with areas where spatial and temporal aggregations of PRRS have occurred (26). Therefore, it is possible that movements, in addition to farm density, might play an important role in the persistent circulation of disease in the area. The co-aggregation of animal movements and PRRS, a disease believed to be transmitted, at least in part, by animal movements (2, 6, 49), suggests that our predicted network might be capturing important features of the underlying industry structure, which indirectly supports the validity of our network predictions.

The characterization of network structures often may help for planning production and designing strategies to control animal disease (7, 8, 11, 18). The approach developed here is an early step for helping in design strategies to control swine diseases regionally. For example, the spread of swine pathogens within the full network can be simulated using computational models, which would be valuable for both predicting patterns of between-farm spread and for evaluating alternate intervention and control strategies. Among them, for instance, vaccination strategies that maximize the collective good could be quantitatively explored, including minimum levels of coverage that may prevent disease circulation in the network. Additionally, the approach developed here may reduce time and cost for data collection, as collection of movements among a partial set of sites might be sufficient to predict movements among a larger set of sites.

Among classification techniques, there are several approaches that might be used to predict possible outcomes, such as links between sites. While the focus of this paper is not to provide an exhaustive review of these techniques, here we offer some ground for further discussion and perhaps comparative studies. The RF approach has high accuracy without overfitting, it is also fairly stable to the presence of outliers and noise, and it may handle the correlation between predictors (40, 41, 53). This may be important in the context of this study, as some atypical movements between sites may occur, predictors may be correlated, and the probability of animal movement between two or more sites may often occur in a non-linear fashion. Alternatively, other supervised techniques might be used. For example, support vector machines, a vector function based technique that splits the data for classification purposes, might resolve non-linearity in the data by using a non-linear kernel function, though its performance sometimes might be compromised (40).

In conclusion, we present an approach to predict the network structure of contacts between and among farms in a region by using partial data. Our results, combined with information on the occurrence of disease in the area (i.e., outbreaks of PRRS within the RCP-N212), may be incorporated into a disease transmission model that will help to evaluate the effectiveness of prevention and control strategies in a region, with the ultimate objective of mitigating the impact of endemic disease and hypothetical epidemic incursions. The approach here may also be applied to other regions and production systems, where information on animal movements is only partially regulated, thus improving decision-makers’ ability to plan and implement disease surveillance and control activities.

## Author Contributions

All the authors have met the four criteria described at the guidelines: PVD designed and data interpretation, revised and approved the version to be published, and agreed to be accountable for all aspects of the work. KV and LJ designed, revised and approved the version to be published, and agreed to be accountable for all aspects of the work. SW data interpretation, revised and approved the version to be published, and agreed to be accountable for all aspects of the work. AP designed, revised and approved the version to be published, and agreed to be accountable for all aspects of the work.

## Conflict of Interest Statement

The authors declare that the research was conducted in the absence of any commercial or financial relationships that could be construed as a potential conflict of interest.

## References

[1] Fèvre EM Bronsvoort BMDC Hamilton KA Cleaveland S Animal movements and the spread of infectious diseases. Trends Microbiol (2006) 14:125–31. 10.1016/j.tim.2006.01.004 16460942 PMC7119069

[2] Albina E. Epidemiology of porcine reproductive and respiratory syndrome (PRRS): an overview. Vet Microbiol (1997) 55:309–16. 10.1016/S0378-1135(96)01322-3 9220627

[3] Dee S Deen J Rossow K Weise C Eliason R Otake S Mechanical transmission of porcine reproductive and respiratory syndrome virus throughout a coordinated sequence of events during warm weather. Can J Vet Res (2003) 67:12–9. 12528824 PMC227022

[4] Dee SA Deen J Otake S Pijoan C An experimental model to evaluate the role of transport vehicles as a source of transmission of porcine reproductive and respiratory syndrome virus to susceptible pigs. Can J Vet Res (2004) 68:128–33. 15188957 PMC1142156

[5] Dee S Clement T Schelkopf A Nerem J Knudsen D Christopher-Hennings J An evaluation of contaminated complete feed as a vehicle for porcine epidemic diarrhea virus infection of naïve pigs following consumption via natural feeding behavior: proof of concept. BMC Vet Res (2014) 10:1–9. 10.1186/s12917-014-0220-9 25091641 PMC4363994

[6] Perez AM Davies PR Goodell CK Holtkamp DJ Mondaca-Fernández E Poljak Z Lessons learned and knowledge gaps about the epidemiology and control of porcine reproductive and respiratory syndrome virus in North America. J Am Vet Med Assoc (2015) 246:1304–17. 10.2460/javma.246.12.1304 26043128

[7] Sydow J Windeler A Organizing and evaluating interfirm networks: a structurationist perspective on network processes and effectiveness. Organ Sci (1998) 9:265–84. 10.1287/orsc.9.3.265

[8] Martínez-López B Perez AM Sánchez-Vizcaíno JM. Social network analysis. Review of general concepts and use in preventive veterinary medicine. Transbound Emerg Dis (2009) 56:109–20. 10.1111/j.1865-1682.2009.01073.x 19341388

[9] Jackson MO Social and Economic Networks. Princeton: Princeton University Press (2008).

[10] Hagerman AD Mccarl BA Carpenter TE Ward MP O’Brien J Emergency vaccination to control foot-and-mouth disease: implications of its inclusion as a U.S. policy option. Appl Econ Perspect Policy (2011) 34:119–46. 10.1093/aepp/ppr039

[11] Nöremark M Håkansson N Lewerin SS Lindberg A Jonsson A. Network analysis of cattle and pig movements in Sweden: measures relevant for disease control and risk based surveillance. Prev Vet Med (2011) 99:78–90. 10.1016/j.prevetmed.2010.12.009 21288583

[12] Rautureau S Dufour B Durand B Structural vulnerability of the French swine industry trade network to the spread of infectious diseases. Animal (2012) 6:1152–62. 10.1017/s1751731111002631 23031477

[13] Büttner K Krieter J Traulsen A Traulsen I. Epidemic spreading in an animal trade network – comparison of distance-based and network-based control measures. Transbound Emerg Dis (2016) 63:e122–34. 10.1111/tbed.12245 25056832

[14] Natale F Giovannini A Savini L Palma D Possenti L Fiore G Network analysis of Italian cattle trade patterns and evaluation of risks for potential disease spread. Prev Vet Med (2009) 92:341–50. 10.1016/j.prevetmed.2009.08.026 19775765

[15] Bajardi P Barrat A Savini L Colizza V. Optimizing surveillance for livestock disease spreading through animal movements. J R Soc Interface (2012) 9:2814–25. 10.1098/rsif.2012.0289 22728387 PMC3479905

[16] VanderWaal KL Picasso C Enns EA Craft ME Alvarez J Fernandez F Network analysis of cattle movements in Uruguay: quantifying heterogeneity for risk-based disease surveillance and control. Prev Vet Med (2016) 123:12–22. 10.1016/j.prevetmed.2015.12.003 26708252

[17] Mardones FO Martinez-Lopez B Valdes-Donoso P Carpenter TE Perez AM The role of fish movements and the spread of infectious salmon anemia virus (ISAV) in Chile, 2007–2009. Prev Vet Med (2014) 114:37–46. 10.1016/j.prevetmed.2014.01.012 24485704

[18] Lentz HHK Koher A Hövel P Gethmann J Sauter-Louis C Selhorst T Disease spread through animal movements: a static and temporal network analysis of pig trade in Germany. PLoS One (2016) 11:e0155196. 10.1371/journal.pone.0155196 27152712 PMC4859575

[19] Shields DA Mathews K Interstate Livestock Movements. Economic Research Service (2003). Available from: http://www.ers.usda.gov

[20] McBride WD Key N U.S. Hog Production from 1992 to 2009: Technology, Restructuring, and Productivity Growth. Washington, DC: US Department of Agriculture, Economic Research Service (2013).

[21] Hurt C Industrialization in the pork industry. Choices (1994) 9:9–13.

[22] Key N McBride W The Changing Economics of U.S. Hog Production, ERR-52. Washington, DC: United States Department of Agriculture, Economic Research Service (2007).

[23] Tilman D Cassman KG Matson PA Naylor R Polasky S. Agricultural sustainability and intensive production practices. Nature (2002) 418:671–7. 10.1038/nature01014 12167873

[24] MacDonald JM McBride WD The Transformation of US Livestock Agriculture: Scale, Efficiency, and Risks. Washington, DC: US Department of Agriculture, Economic Research Service (2009).

[25] USDA. Animal Disease Traceabililty. Washington DC: (2016). Available: https://www.aphis.usda.gov/aphis/ourfocus/animalhealth/SA_Traceability

[26] Valdes-Donoso P Jarvis LS Wright D Alvarez J Perez AM. Measuring progress on the control of porcine reproductive and respiratory syndrome (PRRS) at a regional level: the Minnesota N212 regional control project (Rcp) as a working example. PLoS One (2016) 11:e0149498. 10.1371/journal.pone.0149498 26895148 PMC4760934

[27] Wayne SR Assessment of Demographics and Network Structure of Swine Populations in Relation to Regional Disease Transmission and Control. St Paul, MN: University of Minnesota (2011).

[28] USDA. Census of Agriculture 2012. Minnesota: State and County Data. N.a.S. Service (2014). Available from: http://www.agcensus.usda.gov/Publications/2012/Full_Report/Census_by_State/Minnesota/index.asp

[29] Newman MEJ Mixing patterns in networks. Phys Rev E (2003) 67:1–13. 10.1103/PhysRevE.67.026126 12636767

[30] Wasserman S Faust K Social Network Analysis: Methods and applications. Cambridge: Cambridge University Press (1994).

[31] R Development Core Team. R: A Language and Environment for Statistical Computing. Vienna, Austria: R.F.F.S. Computing (2015).

[32] Wickham H ggplot2: Elegant Graphics for Data Analysis. New York: Springer (2009).

[33] Becker RA Wilks AR maps: Draw Geographical Maps. (2014).

[34] Venables WN Ripley BD Modern Applied Statistics with S. New York: Springer (2002).

[35] Csardi G Nepusz T The igraph software package for complex network research. InterJournal Complex Syst (2006).

[36] Liben-Nowell D Kleinberg J The link-prediction problem for social networks. J Am Soc Inf Sci Technol (2007) 58:1019–31. 10.1002/asi.20591

[37] Lichtenwalter RN Lussier JT Chawla NV New perspectives and methods in link prediction. 16th ACM SIGKDD International Conference on Knowledge Discovery and Data Mining New York, USA (2010).

[38] Newman MEJ. Clustering and preferential attachment in growing networks. Phys Rev E (2001) 64:025102. 10.1103/PhysRevE.64.025102 11497639

[39] Katz L A new status index derived from sociometric analysis. Psychometrika (1953) 18:39–43. 10.1007/BF02289026

[40] Kuhn M Johnson K Applied Predictive Modeling. New York, U S: Springer (2013).

[41] Breiman L Random forests. Mach Learn (2001) 45:5–32. 10.1023/A:1017934522171

[42] Kuhn M Caret: Classification and Regression Training. (2015).

[43] Chen C Liaw A Breiman L Using Random Forest to Learn Imbalanced Data. Berkeley, CA: University of California (2004).

[44] Kuhn M Building predictive models in R using the caret package. J Stat Softw (2008) 28:1–26. 10.18637/jss.v028.i0527774042

[45] Wright MN Ziegler A ranger: a fast implementation of random forests for high dimensional data in C++ and R. arXiv preprint arXiv:1508.04409 [Online] (2015). Available from: https://arxiv.org/abs/1508.04409 (accessed January 11, 2016).

[46] Silverman BW Density Estimation for Statistics and Data Analysis. London: CRC Press (1986).

[47] Duong T ks: kernel density estimation and kernel discriminant analysis for multivariate data in R. J Stat Softw (2007) 21:1–16. 10.18637/jss.v021.i07

[48] Corzo CA Mondaca E Wayne S Torremorell M Dee S Davies P Control and elimination of porcine reproductive and respiratory syndrome virus. Virus Res (2010) 154:185–92. 10.1016/j.virusres.2010.08.016 20837071

[49] Dee S Deen J Burns D Douthit G Pijoan C. An evaluation of disinfectants for the sanitation of porcine reproductive and respiratory syndrome virus-contaminated transport vehicles at cold temperatures. Can J Vet Res (2005) 69:64–70. 15745225 PMC1142172

[50] Giamalva J Pork and Swine. Industry and Trade Summary. Washington, DC: International Trade Commission (2014).

[51] McBride WD Key N Economic and Structural Relationships in U.S. Hog Production. USDA-ERS Agricultural Economic Report – SSRN Electronic Journal (2003).

[52] Alvarez J Valdes-Donoso P Tousignant S Alkhamis M Morrison R Perez A Novel analytic tools for the study of porcine reproductive and respiratory syndrome virus (PRRSv) in endemic settings: lessons learned in the U.S. Porcine Health Manag (2016) 2:1–9. 10.1186/s40813-016-0019-0 PMC5382381 28405429

[53] Liaw A Wiener M Classification and regression by randomForest. R News (2002) 2:18–22. 10.1057/9780230509993

